# Role of the small RNA RyhB in the Fur regulon in mediating the capsular polysaccharide biosynthesis and iron acquisition systems in *Klebsiella pneumoniae*

**DOI:** 10.1186/1471-2180-12-148

**Published:** 2012-07-24

**Authors:** Su-Hua Huang, Chien-Kuo Wang, Hwei-Ling Peng, Chien-Chen Wu, Ying-Tsong Chen, Yi-Ming Hong, Ching-Ting Lin

**Affiliations:** 1 Department of Biotechnology, Asia University, Taichung 41354, Taiwan; 2 Department of Biological Science and Technology, National Chiao Tung University, Hsin Chu 30068, Taiwan; 3 Institute of Genomics and Bioinformatics, National Chung Hsing University, Tai Chung City 40227, Taiwan; 4 Biotechnology Center, National Chung Hsing University, Tai Chung City 40227, Taiwan; 5 Institute of Molecular and Genomic Medicine, National Health Research Institutes, Miaoli County 35053, Taiwan; 6 School of Chinese Medicine, China Medical University, Taichung 40402, Taiwan

**Keywords:** RyhB, Fur, Capsular polysaccharide, Iron acquisition system, *Klebsiella pneumoniae*

## Abstract

**Background:**

The capsular polysaccharide (CPS) and iron acquisition systems are important determinants of *Klebsiella pneumoniae* infections, and we have previously reported that the ferric uptake repressor (Fur) can play dual role in iron acquisition and CPS biosynthesis. In many bacteria, Fur negatively controls the transcription of the small non-coding RNA RyhB to modulate cellular functions and virulence. However, in *K. pneumoniae*, the role played by RyhB in the Fur regulon has not been characterised. This study investigated Fur regulation of *ryhB* transcription and the functional role of RyhB in *K. pneumoniae*.

**Results:**

Deletion of *fur* from *K. pneumoniae* increased the transcription of *ryhB*; the electric mobility shift assay and the Fur-titration assay revealed that Fur could bind to the promoter region of *ryhB*, suggesting that Fur directly represses *ryhB* transcription. Additionally, in a Δ*fur* strain with elevated CPS production, deletion of *ryhB* obviously reduced CPS production. The following promoter-reporter assay and quantitative real-time PCR of *cps* genes verified that RyhB activated *orf1* and *orf16* transcription to elevate CPS production. However, deletion of *ryhB* did not affect the mRNA levels of *rcsA*, *rmpA*, or *rmpA2*. These results imply that Fur represses the transcription of *ryhB* to mediate the biosynthesis of CPS, which is independent of RcsA, RmpA, and RmpA2. In addition, the Δ*fur* strain’s high level of serum resistance was attenuated by the deletion of *ryhB*, indicating that RyhB plays a positive role in protecting the bacterium from serum killing. Finally, deletion of *ryhB* in Δ*fur* reduced the expression of several genes corresponding to 3 iron acquisition systems in *K. pneumoniae,* and resulted in reduced siderophore production*.*

**Conclusions:**

The regulation and functional role of RyhB in *K. pneumoniae* is characterized in this study. RyhB participates in Fur regulon to modulate the bacterial CPS biosynthesis and iron acquisition systems in *K. pneumoniae*.

## Background

*Klebsiella pneumoniae*, a member of *Enterobacteriaceae*, is a rod-shaped gram-negative opportunistic pathogen. A common cause of nosocomial infection, it is also found in various community-acquired infections, including bacteraemia, septicaemia, and urinary tract and respiratory infections, particularly in immunocompromised patients [1-4]. In Asian countries, especially Taiwan and Korea, *K. pneumoniae* is the predominant pathogen found in pyogenic liver abscess in diabetic patients [2,3,5]. The rapid development of antimicrobial resistance in *K. pneumoniae* has further troubled the clinical choices for treatments [6,7]. Studies of the pathogenic mechanisms of *K. pneumoniae* are, therefore, essential in identifying new targets for the development of antibacterial agents.

Multiple virulence factors have been identified to be involved in *K. pneumoniae* infection, which include capsular polysaccharide (CPS), lipopolysaccharides, fimbriae, iron-acquisition system, and antibiotic resistance. Among these factors, CPS is probably considered the major determinants of pathogenesis. The pyogenic liver abscess isolates often carry heavy CPS that could protect the bacteria from phagocytosis and killing by serum factors [8,9]. Apart from the antiphagocytic function, *Klebsiella* CPS also helps the bacterial colonization and biofilm formation at the infection sites [10-12]. The capsular serotypes of *K. pneumoniae* have been classified as more than 77 recognized capsular antigens [13,14]. In Taiwan, a high prevalence of K1 and K2 serotypes of *K. pneumoniae* was documented in liver abscess of diabetes mellitus patients [15]. The *cps* gene clusters that are responsible for the synthesis of different serotypes of CPS have been determined [16]. The K2 *cps* gene cluster of *K. pneumoniae* Chedid contains a total number of 19 open reading frames (ORFs) organized into three transcription units, *orf1-2orf3-15*, and *orf16-17 *[16]. In the previous studies, numerous regulatory systems were demonstrated to control the biosynthesis of CPS via regulating the *cps* transcriptions in *K. pneumoniae*, such as the Rcs system, RmpA, RmpA2, KvhR, KvgAS, and KvhAS [17-20]. Among these, ferric uptake regulator (Fur) represses the gene expression of *rcsArmpA*, and *rmpA2* to decrease CPS biosynthesis [21,22]. Therefore, overlapping regulons governed the regulation of these assorted virulence genes in response to numerous stress conditions.

Bacterial cells are constantly challenged by various environmental stresses from their natural habitats. Similar to many gastrointestinal (GI) pathogens, *K. pneumoniae* faces several challenges during infection and colonisation of the human body. These include gastric acid, the immune system, and a limited supply of oxygen and nutrients [23,24]. Among these, the concentration of iron in the environment is critical for the control of cellular metabolism. Limitation of iron abolishes bacterial growth, but high intracellular concentrations of iron may damage bacteria because of the formation of undesired reactive oxygen species (ROS). Iron homeostasis maintained by the transport, storage, and metabolism of iron is tightly controlled by Fur in many gram-negative bacteria [25-27]. To regulate gene transcription, Fur protein functions as a dimer with Fe^2+^ as a cofactor to bind to a 19-bp consensus sequence, called the Fur box (GATAATGATwATCATTATC; w = A or T), in the promoters of downstream genes [28]. In several gram-negative pathogens, Fur represses the expression of genes involved in iron homeostasis and in the regulation of multiple cellular functions such as oxidative stress, energy metabolism, acid tolerance, and virulence gene production [29-32]. In *K. pneumoniae*, Fur plays a dual role in controlling CPS biosynthesis and iron acquisition [21]. Recently, we also found that type 3 fimbriae expression and bacterial biofilm formation were also controlled by Fur and iron availability [33]. Therefore, the regulatory mechanism of Fur in control of multiple cellular function and virulence factors in *K. pneumoniae* needs to be further investigated.

Although Fur typically acts as a repressor, it also functions as a transcriptional activator for the gene expression such as *acnAfumA*, and *sdhCDAB* (tricarboxylic acid [TCA] cycle enzymes), *bfr* and *ftnA* (iron storage), and *sodB* (iron superoxide dismutase [FeSOD]) [34-38]. However, positive regulation by Fur is often indirect, mediated by Fur-dependent repression of a small non-coding RNA (sRNA), RyhB [39]. RyhB negatively regulates gene expression by base pairing with mRNAs to trigger their degradation via RNase E and RNase III [40]. In many bacteria, RyhB participates in Fur-mediated positive regulation of various important cellular functions, including TCA cycle activity, resistance to oxidative stress, and iron homeostasis in *Escherichia coli* and *Vibrio cholerae *[35,39,41-43]; biofilm formation in *V. cholerae *[44]; and virulence in *Shigella dysenteriae *[45]. In *E. coli*, RyhB has been demonstrated to directly regulate more than 18 transcripts, encoding a total of 56 proteins, most of them involved in iron metabolism [35]. Although the significance of RyhB has been demonstrated in different species, to date, the regulatory relationship of RyhB and Fur, and functionality of RyhB in *K. pneumoniae* has not been studied.

In this study, the regulatory role of Fur in *ryhB* expression in *K. pneumoniae* was investigated. A *ryhB*-deletion mutant in wild type (WT) and Δ*fur* strains and the induced expression of *ryhB* in WT were generated to demonstrate the role of RyhB in mediating CPS biosynthesis and iron acquisition systems.

## Results

### Fur directly represses *ryhB* expression in *K. pneumoniae*

**Figure 1:**
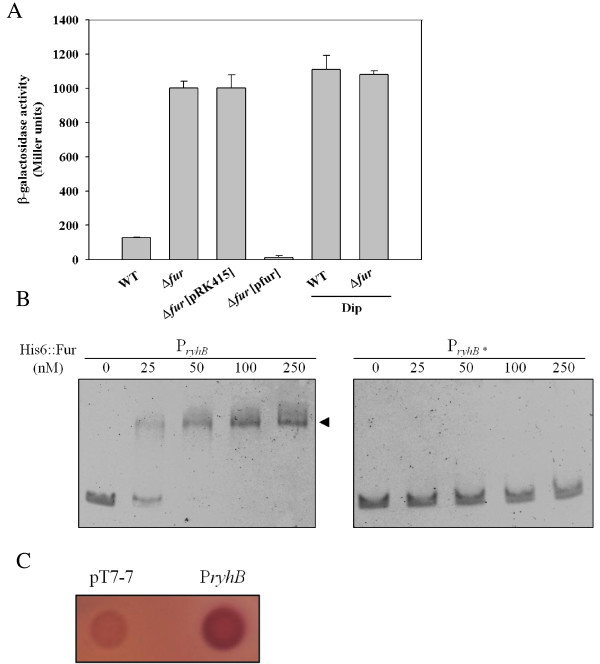
**Fur directly represses the expression of*ryhB*.** (**A**) The β-galactosidase activities of the *K. pneumoniae* CG43S3Δ*lacZ* strain and the isogenic *fur* deletion mutant carrying pRyhB15 (P_*ryhB*_::*lacZ*) were determined from overnight cultures grown in LB with or without Dip. The plasmids pRK415 (vector control) and p*fur* were introduced into Δ*fur* to observe the complement effect. The average of triplicate experiments is shown. Error bars indicate standard deviations. (**B**) EMSA of the recombinant His_6_::Fur and the *ryhB* promoter regions, as indicated in the margin. DNA was incubated with an increasing amount of His_6_::Fur for 30 min, and then loaded onto a 5% non-denaturing polyacrylamide gel. The gel was stained with SYBR Green EMSA stain and photographed. P_ryhB_* indicates deletion of the *fur* box in P_*ryhB*_. (**C**) Assessment of the binding of Fur to the *ryhB* promoter by using the FURTA. *E. coli* H1717 strains carrying the vector control, pT7-7, or the P1 region harboured on pT7-7 are indicated. A red colony (Lac^+^) is considered to have a FURTA-positive phenotype.

To determine whether *K. pneumoniae ryhB* is regulated by Fur, a LacZ reporter system was used. The *ryhB* promoter was cloned into the upstream region of a promoterless *lacZ* gene in placZ15. The resulting plasmid pRyhB15 was then introduced into *K. pneumoniae* CG43S3 Δ*lacZ* and Δ*lacZ*Δ*fur.* The bacterial β-galactosidase activity was measured to assess the expression level of *ryhB*. As shown in Figure 1A, the expression of *ryhB* was higher in Δ*lacZ*Δ*fur* than Δ*lacZ*. Introduction of the complement plasmid p*fur*, but not the empty vector control (pRK415), into Δ*lacZ*Δ*fur* restored the Fur-deletion effect. Moreover, addition of the iron chelator 2, 2-dipyridyl (Dip) to the growth medium increased *ryhB* promoter activity, suggesting that a Fur-Fe(II) complex influences *ryhB* expression. To verify that Fur directly regulates the expression of *ryhB*, an electrophoretic mobility shift assay (EMSA) was performed. As shown in Figure 1B, purified recombinant His_6_-Fur protein was able to bind the upstream region of *ryhB* (P_*ryhB*_), but not the P_ryhB*_ fragment, whose putative Fur-box was deleted. In addition, the binding ability was abolished by the addition of 200 μM EDTA to the reaction mixture (data not shown). Furthermore, *E. coli* H1717, when harbouring a plasmid containing *K. pneumoniae* P_*ryhB*_, also showed a Fur titration assay (FURTA)-positive phenotype (Figure 1C). The results suggest that, in an iron dependent manner, Fur suppresses *ryhB* promoter activity in *K. pneumoniae* by direct interaction with the Fur-box region upstream of *ryhB*.

### RyhB activates CPS biosynthesis

**Figure 2:**
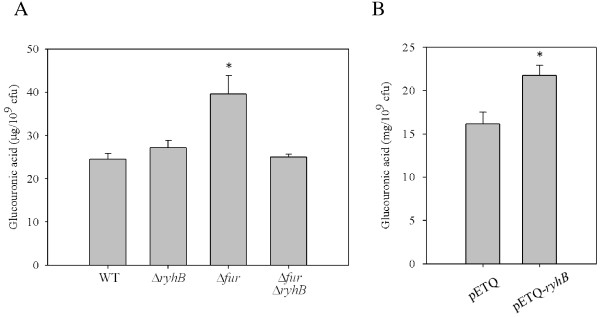
**RyhB activates CPS biosynthesis.** (**A**) Comparison of CPS levels in WT, Δ*ryhB*, Δ*fur*, and Δ*fur*Δ*ryhB* strains. Bacterial strains were grown in LB medium at 37°C with agitation. After 16 h of growth, the bacterial glucuronic acid content was determined. *, *P* < 0.001 compared with WT. (**B**) WT strains carrying the vector control (pETQ) or pETQ-*ryhB* were grown in LB with 100 μM IPTG to induce *ryhB* expression. *, *P* < 0.001 compared with WT strains carrying pETQ.

In *K. pneumoniae* CG43, we found that the deletion of *fur* resulted in elevated CPS production [21,22]. To investigate if RyhB participates in Fur-regulated CPS biosynthesis, the CPS amount was assessed using measuring glucuronic acid content, which served as an indicator for *Klebsiella* K2 CPS [46], in *K. pneumoniae* strains, including WT, Δ*ryhB*, Δ*fur*, and Δ*fur*Δ*ryhB*, was quantified. As shown in Figure 2A, although the deletion of *ryhB* alone did not change on the amount of K2 CPS production, the elevated CPS amount in Δ*fur* cells was abolished by the deletion of *ryhB* when the bacteria were grown in LB medium. The result indicates that Fur regulates the expression of RyhB to repress CPS biosynthesis. To confirm the RyhB expression could activate the CPS biosynthesis, the effect of RyhB induction on CPS amount was determined using an IPTG-inducible vector, pETQ. As shown in Figure 2B, the induced expression of *ryhB* in *K. pneumoniae* CG43 increased CPS production, which confirms that RyhB positively regulates CPS biosynthesis.

### RyhB increased the transcriptional level of the K2 *cps* gene cluster

**Figure 3:**
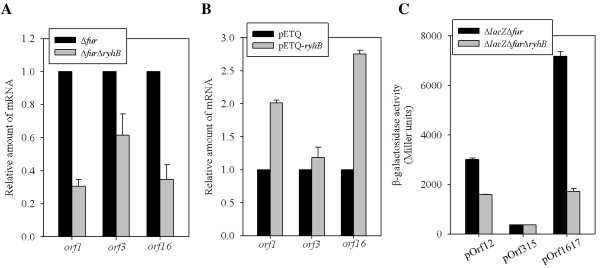
**RyhB activates the transcriptional level of the*orf1*and*orf16*.** (**A**) qRT-PCR analyses of the expression of the K2 *cps* genes (*orf1*, *orf3*, and *orf16*) were measured in Δ*fur* and Δ*fur*Δ*ryhB* strains. (**B**) WT strain carrying the IPTG inducible vector pETQ and pETQ-*ryhB* in response to 100 μM IPTG. (**C**) The β-galactosidase activities of *K. pneumoniae* CG43S3Δ*lacZ*Δ*fur* and Δ*lacZ*Δ*fur*Δ*ryhB* carrying the reporter plasmid pOrf12 (P_*orf1-2*_::*lacZ)*, pOrf315 (P_*orf3-15*_::*lacZ)* or pOrf1617 (P_*orf16-17*_::*lacZ)* were determined using log-phased cultures grown in LB broth. The results shown are an average of triplicate samples. Error bars indicate standard deviations.

To investigate whether RyhB affects the expression of the three *cps* gene clusters, the mRNA levels of *orf1orf3*, and *orf16* in Δ*fur* and Δ*fur*Δ*ryhB* strains were measured by quantitative real-time PCR (qRT-PCR). As shown in Figure 3A, compared to the mRNA levels in the Δ*fur* strain, the mRNA levels of *orf1* and *orf16* were apparent decreased in the Δ*fur*Δ*ryhB* strain, and that of *orf3* also had a slight reduction in the Δ*fur*Δ*ryhB* strain. The result suggests that overexpression of RyhB activated the *cps* gene expression. To confirm our hypothesis, the effect of *ryhB* induction on the mRNA levels of *orf1orf3*, and *orf16* was tested using an IPTG-inducible vector, pETQ. As shown in Figure 3B, the mRNA levels of *orf1* and *orf16* were higher in the pETQ-*ryhB* strain with IPTG induction than the pETQ mock strain, while no significant difference in *orf3* expression was observed. To further investigate whether RyhB acts as a transcriptional activator for the promoter activity of *orf1orf3*, and *orf16*, the reporter plasmids pOrf12 (P_*orf1-2*_::*lacZ*), pOrf315 (P_*orf3-15*_::*lacZ*), and pOrf1617 (P_*orf16-17*_::*lacZ*), each carrying a *lacZ* reporter gene transcriptionally fused to the putative promoter region of the K2 *cps* gene cluster [17], were used to transform the *K. pneumoniae* strains CG43S3Δ*lacZ*Δ*fur* and Δ*lacZ*Δ*fur*Δ*ryhB*. The promoter activity measurements shown in Figure 3C revealed that the deletion of *ryhB* in Δ*lacZ*Δ*fur* reduced activity of P_*orf1-2*_::*lacZ* by at least 50%, while no obvious change was detected in the activity of P_*orf3-16*_::*lacZ*. The activity of P_*orf16-17*_::*lacZ* was reduced by more than 75% in Δ*lacZ*Δ*fur*Δ*ryhB* as compared to the Δ*lacZ*Δ*fur* strain*.* These results imply that RyhB enhances CPS biosynthesis in *K. pneumoniae* by boosting the transcriptional level of the *orf1* and *orf16* gene clusters.

### RyhB does not affect the *rcsA*, *rmpA2*, and *rmpA* mRNA expression level

In previous studies, *K. pneumoniae* Fur was found to repress the expression of genes encoding the *cps* regulatory proteins RcsA, RmpA, and RmpA2 [21,22]. To investigate whether RyhB affects the expression of *rcsArmpA*, and *rmpA2* to increase the *orf1* and *orf16* transcripts, the mRNA levels were measured by qRT-PCR after inducing the expression of *ryhB* in WT. However, qRT-PCR results did not show a significant effect of *ryhB* on the mRNA levels of *rmpArmpA2*, and *rcsA* (Data not shown), suggesting that the activation of RyhB on the *orf1* and *orf16* expression is not via RmpA, RmpA2, and RcsA.

### Deletion of *ryhB* attenuated the higher serum resistance in Δ*fur* strain

**Figure 4:**
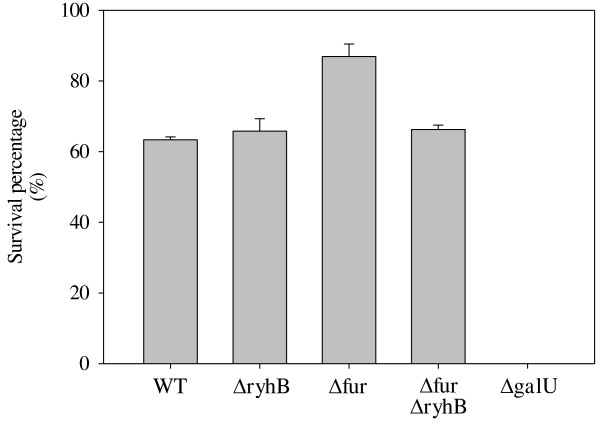
**Effect of Fur and RyhB on susceptibility to normal human serum.** Survival percentage of WT, Δ*ryhB*, Δ*fur*, Δ*fur*Δ*ryhB*, and Δ*galU* (negative control) strains on treatment with 75% healthy human serum was determined, respectively*.* The results shown are an average of triplicate samples. Error bars indicate standard deviations.

In addition to the roles played by RyhB and Fur in regulating the CPS amount, we suggest that RyhB and Fur may also affect the ability of the strain to resist the bactericidal effects of serum. In a human serum resistance assay, we found that the deletion of *fur* in WT increased the survival rate in treatment with 75% normal human serum from 63.3% to 87.9% (Figure 4). However, the deletion of *ryhB* in WT had no apparent effect on the survival rate on treatment with 75% serum, and the higher serum resistance in Δ*fur* cells was abolished by the deletion of *ryhB.* This result indicates that RyhB may participate with Fur in regulating serum resistance in *K. pneumoniae*.

### The regulatory role of RyhB in iron-acquisition systems

**Figure 5:**
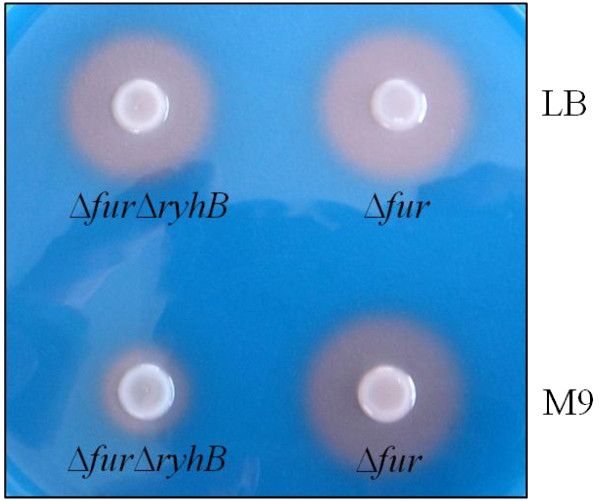
**Deletion of*ryhB*decreases*K. pneumoniae*Δ*fur*siderophore production assessed using CAS assay.** Each of the strains, Δ*fur* and Δ*fur*Δ*ryhB*, was grown overnight in LB medium or M9 minimal medium, and then 5 μl each of cultures respectively was added onto a CAS agar plate. The orange halos formed around the colonies correspond to the iron-chelating activity of the siderophores in bacteria.

To assess whether RyhB affects iron-acquisition in *K. pneumoniae*, the Chrome azurol S (CAS) assay was used to measure siderophore secretions in Δ*fur* and Δ*fur*Δ*ryhB* strains (Figure 5). When bacteria were grown in M9 minimal medium (~2 μM iron) to mimic iron-limited condition, the deletion of *ryhB* in Δ*fur* reduced the formation of the orange halo. However, this change was not observed when bacteria were grown in LB medium (~18 μM iron). Compared to M9 minimal medium contains ~2 μM iron, LB medium is considered an iron-repletion medium. Under iron-repletion, Fur is able to exert its repression on *ryhB* transcription. Thus, *ryhB*-deletion effect is difficult to observed under the growth condition that *ryhB* is poorly expressed. Our results suggest that in the regulation of iron-acquisition systems, RyhB plays a role downstream of Fur in *K. pneumoniae* under iron-limiting conditions.

To investigate the effects on downstream targets of RyhB in iron-acquisition regulons, the expression of genes corresponding to the eight putative iron-acquisition systems in *K. pneumoniae* CG43 was measured in Δ*fur* and Δ*fur*Δ*ryhB* by qRT-PCR (Table 1). In M9 minimal medium, the expression of genes (*iucA*, *fepA*, *fepB*, *entC*, *fecA*, and *fecE*) corresponding to three iron-acquisition systems (aerobactin, enterobactin, and ferric citrate) was decreased by half in the Δ*fur*Δ*ryhB* strain (Δ*fur*Δ*ryhB*/Δ*fur* < 0.5). However, the expression of *fhuA* and *sitA* was significantly increased more than two-fold (Δ*fur*Δ*ryhB*/Δ*fur* > 2.0). These results imply that RyhB activates the expression of *iucA*, *fepA*, *fepB*, *entC*, *fecA*, and *fecE*, but represses the expression of *fhuA* and *sitA*.

**Table 1:** **qRT-PCR analyses of the expression of iron-acquisition genes in*K. pneumoniae*Δ*fur*Δ*ryhB*and Δ*fur*strains**

**Systems**	**Gene**	**RNA expression ratio**^a^
		Δ***fur***Δ***ryhB***/Δ***fur***
Fe^3+^		
Ferrichrome	*fhuA*	2.62 ± 0.07
Aerobactin	*iucA*	0.19 ± 0.06
Enterobactin	*fepA*	0.36 ± 0.01
	*fepB*	0.33 ± 0.05
	*entC*	0.46 ± 0.02
Ferric citrate	*fecA*	0.19 ± 0.02
	*fecE*	0.34 ± 0.03
Salmochelin	*iroB*	0.52 ± 0.05
Heme	*hmuR*	0.69 ± 0.01
Fe^2+^		
Ferrous iron	*feoB*	0.55 ± 0.18
	*sitA*	2.81 ± 0.08

^a^ Mean expression ratio (±SD) of Δ*fur*Δ*ryhB* relative to Δ*fur*.

## Discussion

In this study, we provide an initial characterisation of *K. pneumoniae* RyhB*.* In *K. pneumoniae*, sequence comparison indicated that the nucleotide sequence of the *ryhB* gene (91 bp) is 92.3% identical to the *E. coli* version (90 bp). However, the promoter sequence of *K. pneumoniae ryhB* is only 72.4% identical to that of *E. coli*. In this study, we found that the expression of *ryhB* in *K. pneumoniae* is directly repressed by Fur-Fe(II), as is the case in *E. coli* (Figure 1).

In addition, structure of the genomic neighbourhood of *ryhB* differs between the 2 species. In the *E. coli* genome, *ryhB* is found between *yhhX* and *yhhY*. In the *K. pneumoniae* genome, *ryhB* is flanked by *yhhY* and a hypothetical ORF. By Pfam search, the hypothetical ORF was found to contain a bactofilin domain (E-value = 3.7e-24), which belongs to a new class of polymer-forming proteins that serve as versatile molecular scaffolds in a variety of cellular pathways [47]. Even though the function of this hypothetical protein in *K. pneumoniae* has not yet been investigated, we found that RyhB could strongly repress the expression of this hypothetical protein (unpublished data). This result suggests that RyhB could participate in a variety of cellular pathways in *K. pneumoniae*.

We previously showed in *K. pneumoniae*, Fur represses CPS biosynthesis via regulation of RmpA, RmpA2, and RcsA. In addition to these 3 regulators, one or more regulators may be involved in the Fur-mediated control of *cps* transcription [21]. In this study, we found that RyhB also participates in Fur-regulated CPS biosynthesis via activation of *orf1* and *orf16* transcription and is independent of the 3 regulators, RmpA, RmpA2, and RcsA (Figure 2 and 3). We want to further analyse whether any potential transcriptional regulator-binding motifs exist in the promoter sequences of *orf1* and *orf16*. We noted that a binding site typical of IscR, a transcriptional repressor that controls Fe–S biosynthesis [48], was located 172 bp upstream of the translation start site of GalF (encoded by *orf1,* 5′-ATAACCTGAACGAAAATAAGATTAT-3′). The predication indicated that IscR could participate in control of *orf1* expression. Furthermore, a previous study reported that RyhB promotes the degradation of *iscSUA* transcripts, resulting in an increase in the ratio of apo-IscR/holo-IscR [48]. Whether RyhB activates CPS biosynthesis via regulation of the ratio of apo-IscR/holo-IscR in *K. pneumoniae* awaits further analysis. However, the regulatory mechanism of *cps* transcription is more complex than expected; whether another unknown transcriptional regulator is involved in activation of RyhB’s effect on *orf16* transcription needs to be investigated. In addition, CPS is considered the major determinant that can protect the bacteria from phagocytosis and killing by serum factors [8,9]. In this study, higher serum resistance was found in Δ*fur*, but this higher serum resistance was attenuated by further deletion of *ryhB* (Figure 4). We suggest the protective role of RyhB against serum killing is due to the activation of CPS biosynthesis.

In *E. coli*, RyhB plays a positive role in control of the intracellular iron concentration via the degradation of nonessential iron-using proteins or an increase in siderophore production [49-51]. In this study, we also found the deletion of *ryhB* in Δ*fur* decreased siderophore production on the CAS plate under iron-limiting condition (Figure 5). Consistent with *E. coli *[51], RyhB in *K. pneumoniae* regulates siderophore production by activating the expression of enterobactin system genes (*entCfepA*, and *fepB*). In addition, we found that RyhB may activate *iucA* and *fecA* expression. Since sRNA may positively regulate its target mRNAs via an anti-antisense mechanism to disrupt an intrinsic inhibitory structure in the 5′ mRNA region that sequesters the ribosome-binding site and the first translation codon [52,53], the 5′-untranslated regions of the *iuc* and *fec* operons were analysed for sequences complementary to RyhB by prediction with the bioinformatics application RNAhybrid [54] (http://bibiserv.techfak.uni-bielefeld.de/rnahybrid/submission.html). However, no apparent base pairing was found in the 5′-untranslated region of the *iuc* or *fec* operons, suggesting that the activation of *iucA* and *fecA* by RyhB is not a result of direct interaction. Furthermore, RyhB was found to repress the expression of *fhuA* and *sitA* in *K. pneumoniae*. In *E. coli*, RyhB represses the expression of *fhuA*, which also corresponds to our results [35]. A possible paring between RyhB with the adjacent sequence of translational start site of *fhuA* and *sitA* was also predicted by the RNAhybrid algorithm. Alignment of the protected residues predicts that RyhB forms a 7 + 4 + 4 bp RNA duplex with the *sitA* mRNA (Additional file 1: Figure S1), but no apparent base pairing was found between RyhB and *fhuA*. However, the direct interaction of RyhB with the *sitA* mRNA remains to be confirmed. In *E. coli*, RyhB has been shown to repress several genes that are involved in iron-binding, which may increase the intracellular iron concentration, thereby allowing a better usage of iron and more complete Fur repression of these genes [35,55]. Nevertheless, this possibility in *K. pneumoniae* needs to be proven by careful experiments. In this study, the coordinated action of Fur and RyhB was found to regulate the expression of the iron acquisition systems for maintaining intracellular iron homeostasis in *K. pneumoniae*.

## Conclusions

In this study, we provide an initial characterisation of *K. pneumoniae* RyhB*.* Our results suggest that RyhB plays an important role in the Fur regulon, which modulates the CPS biosynthesis and iron acquisition systems in *K. pneumoniae*, both of which contribute to the infectivity and survival of the bacterium.

## Methods

### Bacterial strains, plasmids, and media

Bacterial strains and plasmids used in this study are listed in Table 2. Primers used in this study are list in Additional file 2: Table S1. Bacterial were routinely cultured at 37°C in Luria-Bertani (LB) medium or M9 minimal medium supplemented with appropriate antibiotics. The antibiotics used include ampicillin (100 μg/ml), kanamycin (25 μg/ml), streptomycin (500 μg/ml), and tetracycline (12.5 μg/ml).

**Table 2:** Bacterial strains and plasmids used in this study

**Strains or plasmids**	**Descriptions**	**Reference or source**
*K. pneumoniae*		
CG43S3	CG43 Sm^r^	[56]
Δ*lacZ*	CG43S3Δ*lacZ*	[17]
Δ*fur*	CG43S3Δ*fur*	[22]
Δ*lacZ*Δ*fur*	CG43S3Δ*lacZ*Δ*fur*	[22]
Δ*ryhB*	CG43S3Δ*ryhB*	This study
Δ*fur*Δ*ryhB*	CG43S3Δ*fur*Δ*ryhB*	This study
Δ*lacZ*Δ*fur*Δ*ryhB*	CG43S3Δ*lacZ*Δ*fur*Δ*ryhB*	This study
Δ*galU*	CG43S3Δ*galU*	[57]
*E. coli*		
DH5α	*supE44* Δ*lacU169 (f80 lacZ*Δμ15) hsdR17 *recA1 endA1 gyrA96 thi-1 relA1*	[58]
BL21-RIL	*F*^*-*^*ompT hsdS*_*B*_*[r*_*B*_^*-*^*m*_*B*_^*-*^*]gal dcm* [DE3]	Laboratory stock
S17-1 *λ pir* H1717	*hsdR recA pro* RP4-2 [Tc::Mu; Km::Tn*7*] [*λpir*] *araD139* Δ*lacU169 rpsL150 relA1 flbB5301 deoC1 ptsF25 rbsR aroB fhuF::λ placMu*	[59,60]
Plasmids		
pKAS46	Positive selection suicide vector, *rpsL* Ap^r^ Km^r^	[59]
yT&A	TA cloning vector	Yeastern
pRK415	Broad-host-range IncP cloning vector, Tc^r^	[61]
pT7-7	Cloning vector, Ap^r^	[62]
pETQ	Km^r^, protein expression vector	[61]
placZ15	Cm^r^, promoter selection vector, *lacZ*^+^	[17]
pfur	Tc^r^, 0.8-kb fragment containing a fur allele cloned into pRK415	[22]
pET30c-Fur	Km^r^, 450-bp fragment encoding full-length Fur cloned into pET30c	[22]
pRyhB04	2.0 kb fragment containing an internal ~70-bp deletion in *ryhB* cloned into pKAS46	This study
pRyhB15	Cm^r^, 178-bp fragment containing the region upstream of *ryhB* cloned into placZ15	This study
pOrf12	Cm^r^, 500-bp fragment containing the region upstream of *Klebsiella K2 cps orf1-orf2* cloned into placZ15	[17]
pOrf315	Cm^r^, 900-bp fragment containing the region upstream of *Klebsiella K2 cps orf3-orf15* cloned into placZ15	[17]
pOrf1617	Cm^r^, 300-bp fragment containing the region upstream of *Klebsiella K2 cps orf16-orf17* cloned into placZ15	[17]
pT7-7-p*ryhB*	178-bp fragment containing the putative *ryhB* promoter, cloned into pT7-7	This study
pETQ-*ryhB*	Km^r^, 326-bp fragment containing the promoter and coding region of *ryhB* cloned into pETQ	This study

### Construction of the gene-deletion mutants

Specific gene deletion was introduced into *K. pneumoniae* CG43S3 using an allelic exchange strategy as previously described [57]. The pKAS46 system was used in the selection of the mutants [59], and the mutations were respectively confirmed by PCR and Southern hybridization (data not shown).

### Measurement of promoter activity

The promoter region of *ryhB* was PCR-amplified with primer pair pGT44/pGT45, and the amplicons were then cloned into placZ15 [63]. The promoter-reporter plasmids, pRyhB15, pOrf12, pOrf315, and pOrf1617, were individually mobilized into *K. pneumoniae* strains by conjugation from *E. coli* S17-1 λ*pir*. The bacteria were grown to logarithmic phase in LB broth with or without 200 μM Dip (OD_600_ of 0.7), and the β-galactosidase activity was measured as previously described [63].

### EMSA

Recombinant *K. pneumoniae* Fur protein was expressed in *E. coli* and purified as previously described [22]. DNA fragment of the putative promoter region of *ryhB* was respectively PCR amplified by using specific primer sets (Table 2). The purified His_6_-Fur was incubated with 10-ng DNA in a 15 μl solution containing 50 mM Tris–HCl (pH 7.5), 100 mM NaCl, 100 mM dithiothreitol, 200 μM MnCl_2_, and 1 μg/μl BSA at room temperature for 20 min. The samples were then loaded onto a native gel of 5% nondenaturing polyacrylamide containing 5% glycerol in 0.5× TB buffer (45 mM Tris–HCl, pH 8.0, 45 mM boric acid). Gels were electrophoresed with a 20-mA current at 4°C and then stained with SABR safe Gel stain (Invitrogen).

### FURTA

FURTA was performed according to the method described by Stojiljkovic *et al. *[64]. DNA sequences containing a putative Fur box were PCR amplified with specific primer sets and then cloned into pT7-7. The resulting plasmids were introduced into the *E. coli* strain H1717, and the transformants were plated onto MacConkey-lactose plates containing 100 μg/ml ampicillin and 30 μM Fe(NH_4_)_2_(SO_4_)_2_. The indicator strain H1717 contained a chromosomal *fhuF::lacZ* fusion, and a low affinity Fur box has been demonstrated in the *fhuF* promoter. The introduction of pT7-7 derived plasmids carrying Fur-binding sequences could thus cause the removal of Fur from the *fhuF* Fur box [60]. H1717 harboring pT7-7 was used as a negative control. Colony phenotype was observed after incubation at 37°C for 10 h. Red colony (Lac+) denoted a FURTA-positive phenotype and indicated the binding of Fur to the DNA sequence cloned into the pT7-7 plasmid.

### Extraction and quantification of CPS

CPS was extracted and quantified as previously described [65]. The glucuronic acid content, represents the amount of *K. pneumoniae* K2 CPS, was determined from a standard curve of glucuronic acid (Sigma-Aldrich) and expressed as micrograms per 10^9^ CFU [46].

### qRT-PCR

Total RNAs were isolated from early-exponential-phase grown bacteria cells by use of the RNeasy midi-column (QIAGEN) according to the manufacturer’s instructions. RNA was DNase-treated with RNase-free DNase I (MoBioPlus) to eliminate DNA contamination. RNA of 100 ng was reverse-transcribed with the Transcriptor First Strand cDNA Synthesis Kit (Roche) using random primers. qRT-PCR was performed in a Roche LightCycler® 1.5 Instrument using LightCycler TaqMan Master (Roche). Primers and probes were designed for selected target sequences using Universal ProbeLibrary Assay Design Center (Roche-applied science) and listed in Additional file 2: Table S1. Data were analyzed using the real time PCR software of Roche LightCycler® 1.5 Instrument. Relative gene expressions were quantified using the comparative threshold cycle 2^-ΔΔCT^ method with 23S rRNA as the endogenous reference.

### Bacterial survival in serum

Normal human serum, pooled from healthy volunteers, was divided into equal volumes and stored at −70°C before use. Bacterial survival in serum was determined with minor modifications [57]. First, The bacteria were grown to log phase in LB broth and the viable bacterial concentration was adjusted to 1 × 10^6^ colony forming units/ml. 1 ml of the cultures was washed twice by using phosphate-buffered saline (PBS) and resuspended in 1 ml PBS. The mixture containing 250 μl of the cell suspension and 750 μl of pooled human serum was incubated at 37°C for 60 min. The number of viable bacteria was then determined by plate counting. The survival rate was expressed as the number of viable bacteria treated with human serum compared to the number of pre-treatment. The assay was performed triple, each with triplicate samples. The data from one of the representative experiments are shown and expressed as the mean and standard deviation from the three samples. The 0% survival of *K. pneumoniae* CG43S3Δ*galU* served as a negative control.

### CAS assay

The CAS assay was performed according to the method described by Schwyn and Neilands [66]. Each of the bacterial strain was grown overnight in M9 minimal medium, and then 5 μl of culture was added onto a CAS agar plate. After 24 hr incubation at 37°C, effects of the bacterial siderophore production could be observed. Siderophore production was apparent as an orange halo around the colonies; absence of a halo indicated the inability to produce siderophores.

### Statistical method

An unpaired *t*-test was used to determine the statistical significance and values of *P* < 0.001 were considered significant. The results of CPS quantification and qRT-PCR analysis were derived from a single experiment representative of three independent experiments. Each sample was assayed in triplicate and the mean activity and standard deviation are presented.

## Competing interests

The authors declare that they have no competing interests.

## Authors’ contributions

SHH, CKW, HLP, and CTL made substantial contributions to design and conduct the experiments. YMH performed qRT-PCR and growth experiments. SHH and CKW performed the bioinformatics analyses and interpretation of data. CCW, YTC, and HLP contributed to the writing and editing of the manuscript. CTL coordinated the study and performed manuscript editing. All authors have read and approved this work.

## Supplementary Material

Additional file 1: Figure S1 RyhB pairs with *sitA*. The file contains supplemental figure S1 that the potential base pairing in RyhB/*sitA* mRNA in this study. Click here for file

Additional file 2: Table S1 Primers used in this study. The file contains supplemental Table S1 that the detailed information of primer sets used in this study. (DOC 64 kb) Click here for file
