# Supplementary figures and images for: Role of the small RNA RyhB in the Fur regulon in mediating the capsular polysaccharide biosynthesis and iron acquisition systems in Klebsiella pneumoniae
Source: BMC Microbiol. 2012 Jul 24;12:148. doi: 10.1186/1471-2180-12-148 (PMC3423075; doi:10.1186/1471-2180-12-148)

## Slide 1
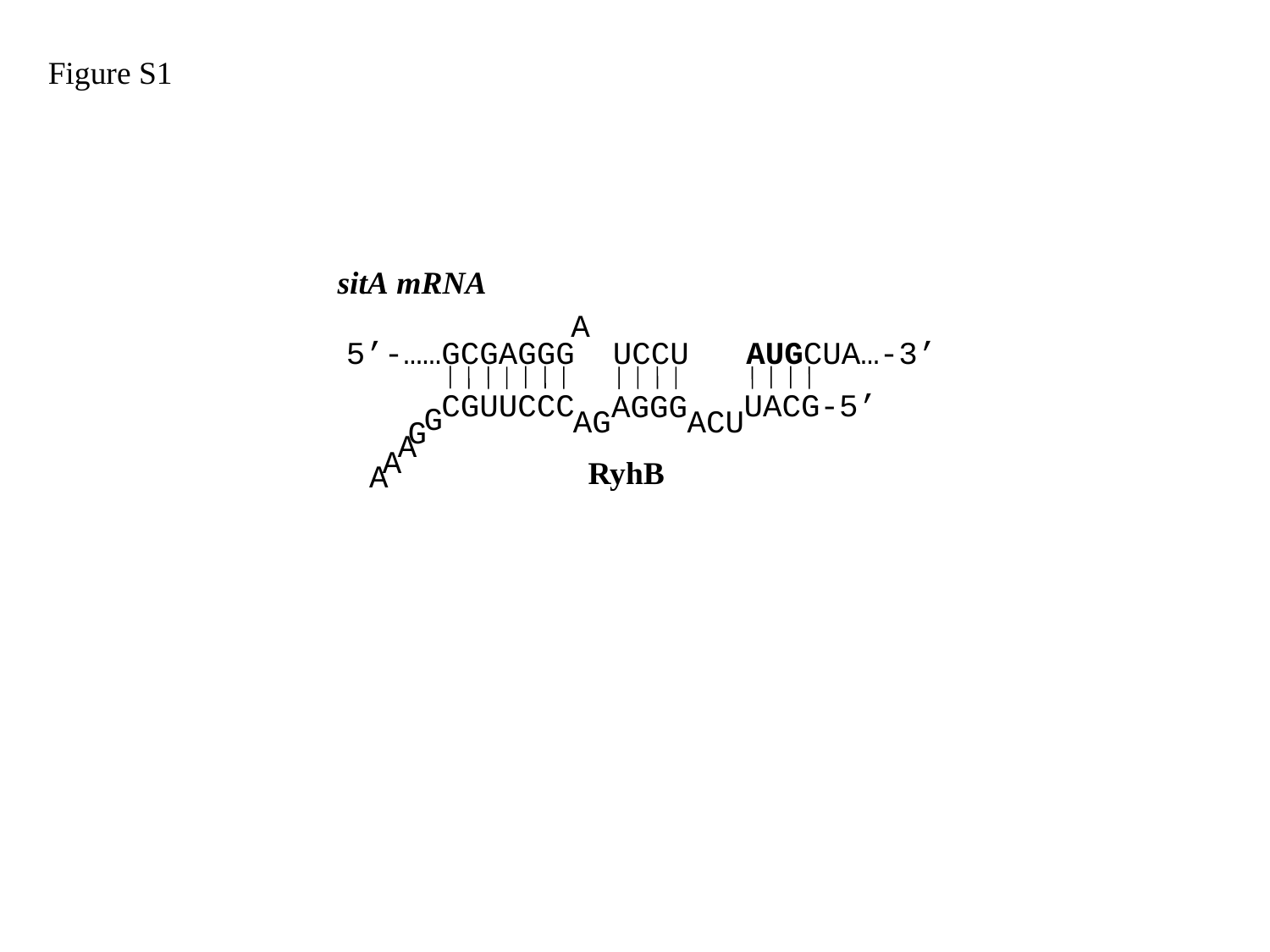

Figure S1
sitA mRNA
A
CGUUCCC
UACG-5’
AGGG
G
ACU
AG
G
A
A
RyhB
A
5’-……GCGAGGG UCCU AUGCUA…-3’

Supplement: Additional file 1: Figure S1 — RyhB pairs with sitA. The file contains supplemental figure S1 that the potential base pairing in RyhB/sitA mRNA in this study. [file 1471-2180-12-148-S1.ppt]
