# Supplementary material for: Role of the small RNA RyhB in the Fur regulon in mediating the capsular polysaccharide biosynthesis and iron acquisition systems in Klebsiella pneumoniae
Source: BMC Microbiol. 2012 Jul 24;12:148. doi: 10.1186/1471-2180-12-148 (PMC3423075; doi:10.1186/1471-2180-12-148)
Supplement: Additional file 2: Table S1 — Primers used in this study. The file contains supplemental Table S1 that the detailed information of primer sets used in this study. (DOC 64 kb) [file 1471-2180-12-148-S2.doc]

**Table S1 Primers used in this study**

| Primer | Sequence (5’🡪3’) | Enzyme cleaved | |
| --- | --- | --- | --- |
| YCC015 | tctagaCGCTGTCGTCAATGTCGC | *Xba*I | |
| YCC016 | TCCGCAAAGATCTCCCTGAATG | *Bgl*II | |
| YCC017 | TTTTGCTGAGATCTCAGGCG | *Bgl*II | |
| YCC018 | CTTAGTTAATGAGCTCCCTTC | *Sac*I | |
| GT11 | ggatccCTCGGTCTGGTAGCAGACGC | *Bam*HI | |
| GT12 | aagcttCGGTTCAGCATGGCGTATC | *Hin*dIII | |
| GT44 | ggaTCCGCAAGGGTCTCCCTG | *Bam*HI | |
| GT45 | agatctCGGTTCAGCATGGCGTATC | *Bgl*II | |
| GT111 | GGTGGATCCTCTCGCGCATCA | *Bam*HI | |
| For RT-qPCR | Sequence (5’🡪3’) | TaqMan probes | Target |
| GT56 | accccgccagctttaactt | 3 | *entC* |
| GT57 | tgtccttctttacgcagcag |
| GT58 | caacctgaacagcgatttcc | 20 | *fecA* |
| GT59 | tcggcgctctctttaacagt |
| GT62 | cagatgtcagcgcagatcc | 20 | *feoB* |
| GT63 | cataggcccggctgtaga |
| GT64 | aaagagattggcctcgagttt | 20 | *fepA* |
| GT65 | tgttgcggtagtcgttgc |
| GT66 | aataaacagctcgtttcgttaaaag | 160 | *fepB* |
| GT67 | gtatagaccagggcggtcac |
| GT68 | gtttggtcgtatcgcctgac | 3 | *fhuA* |
| GT69 | ggaaggtgaagtcagttttatcg |
| GT72 | tgatgacctacctgcagtacca | 20 | *hmuR* |
| GT73 | gagccgaggttccaggag |
| GT74 | cggaggaacattcgtcaaa | 84 | *iroB* |
| GT75 | ttcggaatctaagcctggtg |
| GT78 | tctcccggcttattgttgata | 67 | *iucA* |
| GT79 | ggaaggtttcgcaactggt |
| GT82 | gaagatccgtcagacgatgg | 20 | *sitA* |
| GT83 | tagtcgcgggccagatag |
| RT03 | cgtcatccagaccaaagagc | 83 | *orf1* |
| RT04 | ccggtttttcaataaactcgac |
| RT05 | cgatgaccggctttttaatg | 83 | *orf3* |
| RT06 | ctagcggagatttggtactgc |
| RT07 | cagtccacctttattccgattg | 67 | *orf16* |
| RT08 | aggtacgaccccgactgg |
| RT11 | ggtaggggagcgttctgtaa | 67 | 23S rRNA |
| RT12 | tcagcattcgcacttctgat |
| RT17 | tcaatagcaattaagcacaaaagaa | 18 | *rmpA* |
| RT18 | ttgtaccctccccatttcc |
| RT19 | aaatcattacccacaactaacaaaaa | 80 | *rmpA2* |
| RT20 | ttagacggctttttaattcatgg |
| GT25 | aaaacagaatcaaatatgctgcaa | 158 | *rcsA* |
| GT26 | cgttgagatttgcgaagtacc |
